# Unmasking identity dissonance: exploring medical students’ professional identity formation through mask making

**DOI:** 10.1007/s40037-017-0339-z

**Published:** 2017-02-28

**Authors:** Kimera Joseph, Karlen Bader, Sara Wilson, Melissa Walker, Mark Stephens, Lara Varpio

**Affiliations:** 1 Uniformed Services University of the Health Sciences (USU), Bethesda, MD USA; 2 Department of Family Medicine, University of the Health Sciences (USU), Bethesda, MD USA; 3 National Intrepid Center of Excellence, Walter Reed National Military Medical Center, Bethesda, MD USA

**Keywords:** Mask making, Professional identity formation, Identity dissonance, Medical education

## Abstract

**Purpose:**

Professional identity formation is an on-going, integrative process underlying trainees’ experiences of medical education. Since each medical student’s professional identity formation process is an individual, internal, and often times emotionally charged unconscious experience, it can be difficult for educators to understand each student’s unique experience. We investigate if mask making can provide learners and educators the opportunity to explore medical students’ professional identity formation experiences.

**Methods:**

In 2014 and 2015, 30 third year medical students created masks, with a brief accompanying written narrative, to creatively express their medical education experiences. Using a paradigmatic case selection approach, four masks were analyzed using techniques from visual rhetoric and the Listening Guide.

**Results:**

The research team clearly detected identity dissonance in each case. Each case provided insights into the unique personal experiences of the dissonance process for each trainee at a particular point in their medical school training.

**Conclusions:**

We propose that mask making accompanied by a brief narrative reflection can help educators identify students experiencing identity dissonance, and explore each student’s unique experience of that dissonance. The process of making these artistic expressions may also provide a form of intervention that can enable educators to help students navigate professional identity formation and identity dissonance experiences.

## What this paper adds

Professional identity formation is an essential element of each trainee’s medical education. Medical students endure many challenges related to professional identity formation that are easily undetected amidst other required training objectives. This paper adds an uncommon modality to assist in the exploration of a student’s ongoing professional identity formation process. By using mask making as a means of personal expression, educators may be able to further understand how students identify themselves as well as to assist medical students as they navigate the process of professional identity formation.

## Introduction

The process of professional identity formation in medical education is an area of active investigation [1–5]. Professional identity formation is an on-going, dynamic process through which a medical trainee navigates and takes on the identity and roles of a physician [1]. As a medical student learns to become a physician, she/he engages in the basic and essential process [3] of integrating ‘the knowledge, skills, values and behaviours of a competent, humanistic physician with [her/his] own unique identity and core values’ [6]. Students navigate the process of integrating their new identity as a physician with individual sub-identities within the competitive and demanding environment of medical education.

For some medical students, negotiating the processes of professional identity formation is relatively straightforward since their personal identities neatly align with those of their new professional role [7]. In contrast, for trainees whose personal and professional identities are not congruent, professional identity formation can be a distressing process that requires them to adopt an altered world-view with different values and emotional orientations [3]. This incongruity has been termed identity dissonance [7]: the disconcerting internal experience of conflict between irreconcilable aspects of self [8]. Identity dissonance occurs when individuals encounter difficulty as they seek to incorporate the identity of a physician with former established identities [1, 3, 7, 9]. Identity dissonance can be a significant emotional disruption and can contribute to students questioning their values, ambitions, abilities, and ‘their very self-worth’ [7, p. 26]. Students most likely to experience dissonance are those who feel that they do not belong as part of the medical community and those who reject alterations of their existing identity [1]. Students who avoid the formation and integration of their personal and professional identities often struggle to find success in their chosen field [7].

To better understand emotional experiences involved with professional identity formation, including identity dissonance, Monrouxe suggests that scholars should broaden their methods of data collection and analysis and take a deeper look at the processes involved [3]. In response to that call, we report on the use of mask making techniques to help students examine aspects of their individual, internal emotional experiences related to medical training. This exploratory qualitative study began by asking if mask making, accompanied by a short reflective narrative, could facilitate the expression of personal identity. As the analysis progressed, we realized that some students were harnessing the mask making exercise as a means to express their professional identity formation processes, particularly elements related to identity dissonance. In this paper, we explore 1) how learners expressed identity dissonance in their masks and accompanying narratives and 2) what could we learn about each learner’s unique experience of identity dissonance through these expressions.

## Methods

Working from an interpretivist orientation [10], we used an exploratory multiple-case study research design [11]. We began with exploratory data collection and narrowed our research focus to identity dissonance [12]. The Uniformed Services University of the Health Sciences (USU) approved the collaboration with the National Intrepid Center of Excellence for the making of the masks and narratives. Approval for the study was obtained through the USU Institutional Review Board.

### Setting

Our study was conducted at the USU, the only federal medical school in the United States (US). USU students are commissioned officers in the US military or US Public Health Service who serve as active duty officers throughout their education. After completing their medical education (including residency), graduates return an obligated minimum of seven years of service as medical officers.

### Intervention: mask making

Mask making is an art practised since the early days of civilization by societies and cultures around the world. Masks are acknowledged forms of communication that allow for the external expression of concerns residing within the individual [13]. Masks provide a glimpse into the boundaries of identity and enable individuals to explore personal meanings and feelings of belonging [14, 15]. Furthermore, mask making has been employed as art therapy and as a psychotherapeutic modality using ‘the creative process, and the resulting artwork to explore [clients’] feelings, reconcile emotional conflicts, [and] foster self-awareness’ [16]. Art therapists laud mask making for enabling an individual to explore his/her persona, portraying both ‘how others see you’ and ‘how you really feel inside’ [17]. Thus, art therapy research suggests that mask making supports the exploration of personal identity [18]. It should be noted, however, that art therapy research has been critiqued since much of the literature relies on poorly described interventions, with small participant populations, and using participant self-assessed emotional improvement as a prime measure of efficacy [19, 20]. Despite these criticisms of art therapy, we felt that mask making offered an innovative approach to exploring medical students’ professional identity formation experiences and warranted rigorous investigation. Professional identity formation and identity dissonance are deeply personal, internal experiences. Since mask making can support the exploration of identity and personal meanings, the process of mask making is uniquely positioned to provide insight into professional identity formation and identity dissonance. Indeed, mask making allows students to bridge ‘the gap between the conscious and the unconscious, often providing a depth of clarity, understanding, and empathy otherwise difficult to achieve through words alone’ [21, p. 959].

We realize that masks are non-linguistic forms of creative expression. Other research has shown how the artistic expressions that medical students create offer visual evidence of how their experiences affect their professional identity formation [22]. In that research, comics were used to help medical students reflect on their experiences while providing an outlet to express their concerns [22]. Importantly, comics use linguistic and non-linguistic expressions to convey meanings. We decided that mask making might usefully benefit from accompanying artist-made narratives to provide the artists with additional means of expression.

### The mask-making process

In 2014 and 2015, third year medical students were recruited via an email invitation to voluntarily participate in a mask making session that was not part of the graded curriculum. The session was held at the National Intrepid Center of Excellence (NICoE), located next to USU. NICoE’s art therapy mask making program has been recognized for helping wounded war veterans express the psychological pain associated with combat [23]. The lead therapist of this program (MW) in collaboration with a USU faculty member (MS) felt that we could borrow some techniques from the program to support USU medical students.

Students were informed that the purpose of the session was to produce a mask to creatively express their medical education experiences. During the session, they were introduced to the concept of art therapy and given examples of how the treatment is used with wounded warriors [16, 23]. The students engaged in a short group discussion about medical school and the importance of taking time for self-care. Each student was then given a blank papier-mâché mask and was invited to symbolize her or himself within the mask. Students had an array of art supplies to use in this activity including acrylic paint, air-drying clay, markers, pencils, glitter, glue, feathers, beads, and other found objects. The students worked on the masks for approximately 90 min, and were then asked to reflect on and write about their work. These hand-written reflections were completed in approximately 15 min. At the session’s end, students were invited to share the meaning behind their masks, allowing for group processing. The art therapist did not disclose the content of the group discussion with the analysts.

### Case selection

Thirty students participated in the mask making activity. In 2015, two investigators (MS and LV) viewed all the masks and accompanying narrative reflections looking for common attributes across the artifacts. Following a paradigmatic case selection approach [24], these investigators used criterion sampling [25] to select masks and accompanying narratives that they felt depicted and described the participant’s identity. To make these determinations, the investigators read the personal narratives looking for expressions of identity (e. g., statements of ‘I am’, ‘myself’, ‘my role’, etc.) and examined the masks for visual representations of identity (e. g., visual expressions of personal tension such as masks decorated in different ways on different sides of the face; of medical identity such as a caduceus and of military identity such as camouflage paint or thematic service emblems). The investigators selected eight submissions that they felt depicted each participant’s identity and presented them to the full research team as possible cases for the study. The team examined these masks and narratives, and discussed their potential to offer rich insights into identity. The team unanimously agreed on four masks and their accompanying narratives to include as the cases for this study. Serendipitously, female students created all four cases.

### Data analysis

We analyzed each case individually, using the Listening Guide to analyze the narrative and visual rhetoric to analyze the mask. This combination of analytical approaches has been successfully used to interpret artistic creations by other health professional trainees [26]. The Listening Guide was developed by Dr. Carol Gilligan to analyze the multiple voices present in an individual’s statements (including, for example, voices of cultural context, relationships with others, perceptions of self, political views, etc.) [27]. Listening Guide’s four steps enable the analyst(s) to hear and analyze multiple voices contained within a single utterance [27]. These four steps are: listening for plot, creating i‑poems, listening for contrapuntal voices, and composing analysis. In each step, the analyst listens for different elements of voice being expressed (or silenced) in the statement to hear the complexity of the individual’s experiences and contexts. Listening Guide’s approach recognizes how the analyst is actively participating in identifying and interpreting the voices in the statements. This interpretivist orientation is congruent with our research design.

Visual rhetoric examines the visual designs in artifacts to identify and analyze the culturally specific messages conveyed by the artist. We used Kress and van Leeuwen’s visual grammar [28], specifically the compositional structures of Given/New and Saliency, to understand how visual conventions produce specific meanings by the student-artists. Given/New distinction addresses the socially different values placed on the left/right sides of a visual image. The left is Given, meaning that the visual elements on the left are ‘presented as something the viewer already knows, as familiar’ [28, p. 187]. The right is New, meaning that visual elements on the right are ‘presented as something which is not yet known, or perhaps not yet agreed upon’ [28, p. 187]. Saliency is the degree to which a visual element draws attention to itself (e. g., via differences in size, colour, foregrounding, etc.). Visual rhetoric rests on the understanding that meaning making is culturally and individually specific. In other words, visual rhetoric acknowledges that individual viewers can interpret artistic expressions differently. This orientation makes visual rhetoric compatible with our interpretivist research design.

One investigator (LV), who is trained in and has used visual rhetoric and the Listening Guide extensively in research, trained the analysis team in these approaches, participating in all activities, and ensuring that the team adhered to visual rhetoric and Listening Guide analysis procedures. For each case, the analysis team members (KJ, KB, SW, MW and LV) independently analyzed the case using the Listening Guide and visual rhetoric. The team then met to discuss the case, reflect on similarities and differences in interpretations, and collaboratively constructed a summary of the analyses. For each mask, we used Cristancho et al.’s audit trail for aesthetic analysis [29] to compile the group’s visual rhetoric-based interpretations. For each narrative reflection, the summary consisted of a complete description of the group’s Listening Guide analysis. While the analysis of each case generated many themes, those related to professional identity formation and expressions of identity dissonance were particularly resonant across the selected cases.

Given this study’s interpretivist orientation, we acknowledge that the research team actively participated in creating the findings presented in this study. To support trustworthiness via reflexivity, we discussed our individual research perspectives, positions, and histories to consider how our personal contexts influenced our analyses. To be transparent about these influences, the biographical notes at the end of the text briefly summarize the pertinent personal contexts of each analyst. We also maintained an audit trail of all study processes to support confirmability.

## Results

**Fig. 1:**
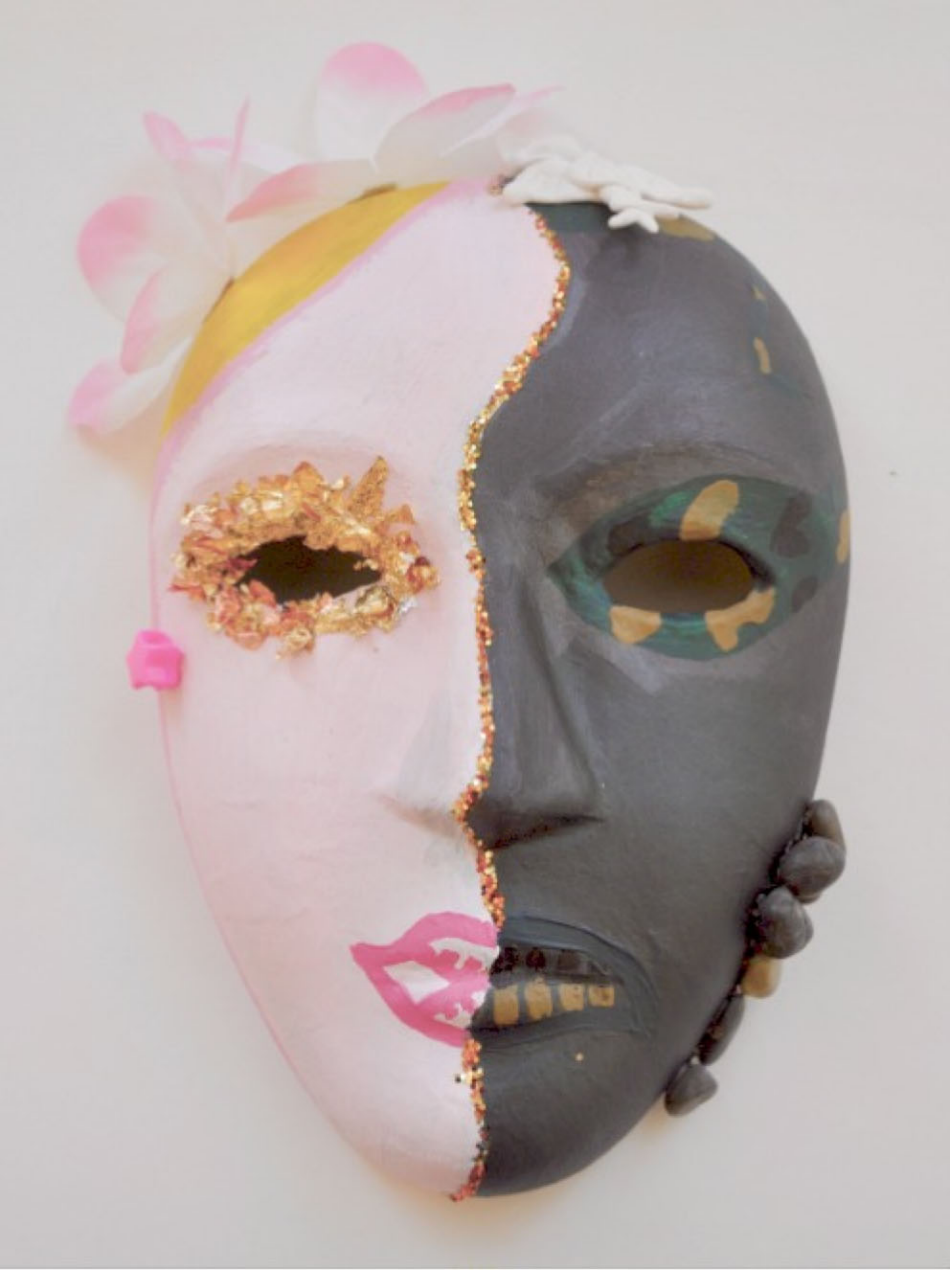
Fictional mask

**Fig. 2:**
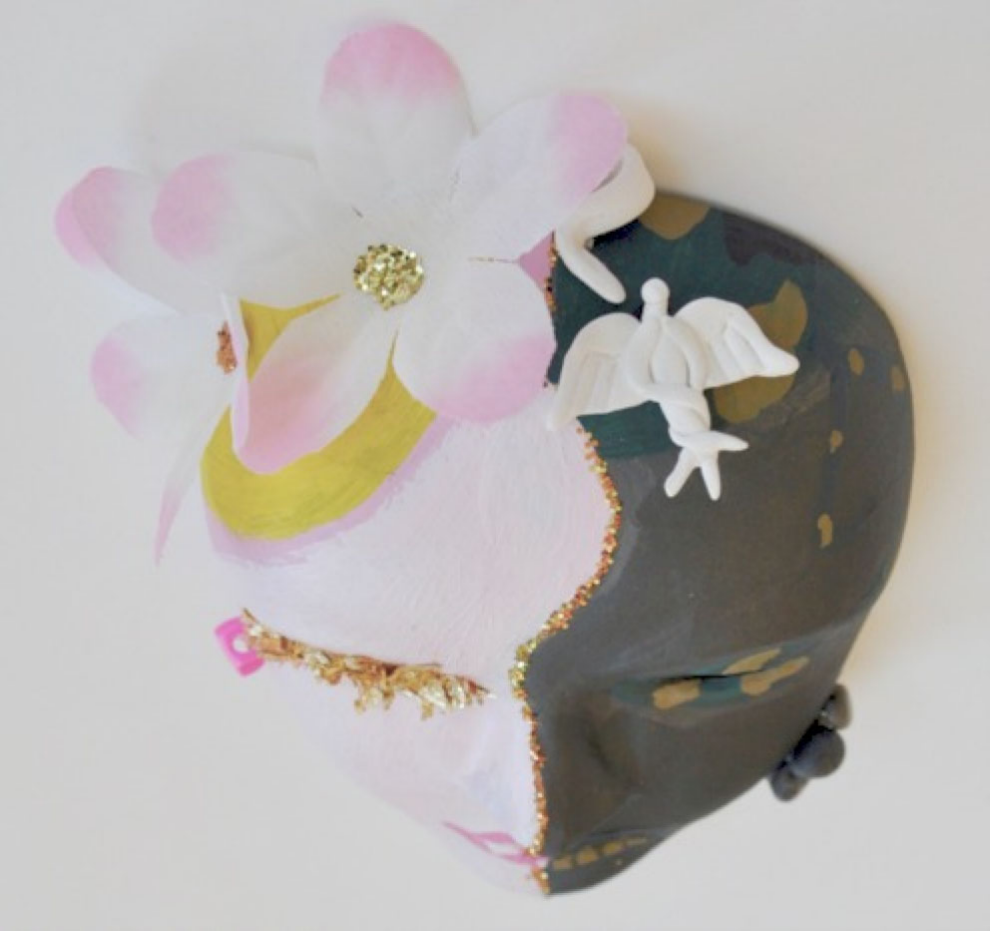
Top of fictional mask

**Fig. 3:**
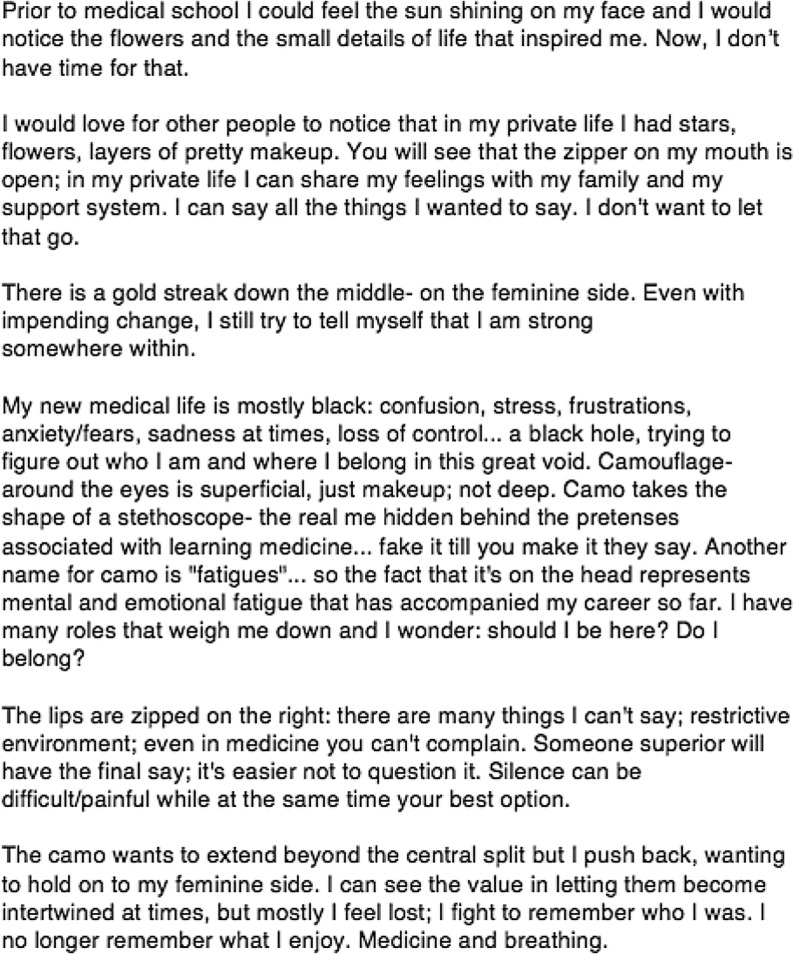
Fictional narrative

The following series of case reports describes how the analysis team recognized identity dissonance in each case. Each case is highly personal and reveals private, sensitive participant reflections. To preserve the anonymity of the participants, we provide only excerpts from each case. To illustrate a whole case, we created a mask and narrative of a fictional student to reflect the identity dissonance themes observed in this study’s four cases (Figs. 1, 2 and 3).

### Case A

In this case, the student’s experience of identity dissonance is evident in the narrative. She lists the many roles that are expected of her, and laments losing her ‘own self’ behind these multiple expectations. This role burden is most evident in the i‑poem found in the beginning of the narrative:

I am a mom,a Marine,a wife,a Soldier,a sister,an Engineer,a daughter,a student-doctor,a neighbour,a college student,a carpool mom,an artistmy own self gets lost behind all of these things.

We heard this student’s bewilderment in the loss of self, and in the pulls that these roles exert on her. She expresses how the tension across these roles is exacerbated by the new and weighty expectations of being a medical student. We heard her feeling lost and unable to sew together a complete identity that is recognized by others (‘I am not who you think I am,’ ‘It hurts to not be recognized’). We heard her descriptions of feeling personally and professionally inadequate, unable to fully engage in each role. Across the narrative, we heard this student’s struggle to advance in integrating her professional physician identity. We heard her frustration at being unable to embrace and embody all of her roles, including as a military physician.

Her mask visually depicts the tension she experiences from her multiple roles and responsibilities. The face is vertically split into two halves. The left, Given side, is painted pink, decorated with a floral pattern and glitter. The right, New side, is painted with the military’s camouflage pattern. The mask visually portrays the student’s feminine side as the familiar, primary identity, while the military identity is contested, and not yet agreed upon. There is a single, beige-coloured puzzle piece on the forehead, connecting the two halves. In contrast to the two colourful sides of the mask, the puzzle is a visually salient element. We interpreted this as a representation of the incomplete connection – the missing puzzle piece – the student needs to unite her distinct roles.

In sum, we heard this student’s expressions of being in the throes of identity dissonance. Her narrative resonates with pain and the struggle to bring her primary identity and new professional identity into harmony.

### Case B

In this case we again hear a female student wrestling with identity dissonance. We were struck by the change in voice in the narrative’s i‑poem. The student used the personal pronoun ‘my’ in association with the mask’s portrayal of life prior to medical school:

my complexion is rosymy eyes are brightmy lips turn upward in a smilemy mental landscape is represented by a shining sun

While describing being in medical school, she no longer represents herself in the descriptions of the mask. In describing her life at USU, she uses the definite article ‘the’ instead of personal pronouns to refer to her physical self:

the lips drawn togetherthe eyes more plainthe cheeks less rosy.

In this linguistic transition, we heard the silenced gap that separates the student’s personal identity (i. e., my) from her professional identity as a military medical trainee (i. e., the).

We also identified this tension in the visual differences between the left and right sides of the mask. On the left, Given side, her lips are brightly painted, upturned and smiling. Yet on the right, New side, her lips are plain, drawn together and serious. On the left, Given side, a yellow sun shines from the top left of the mask. On the right, New side, grey bricks sit on top of the mask and camouflage face paint streaks across her cheek. Collectively, the mask visualizes the divide between the joy and hope this student held within her primary identity and the professional identity she resists of the austere, military context.

Like in Case A, this student expresses identity dissonance in terms of the different and incongruent emotional orientations that her primary and professional identities demand.

### Case C

This case reveals a different experience of the identity dissonance processes. While listening for plot, we heard a significant change in voice and action between the beginning and end of the narrative. This case’s narrative begins with the student’s descriptions of being stagnant in her development as a military officer, and of determination to hold on to her feminine identity. She describes her mask as ‘a reflection of me in my transition from the civilian world to the military world.’ She writes that ‘my progress in transitioning has not changed much’ and ‘my military bearing has not improved.’ She describes her femininity as a part of her identity that she refuses to lose, stating ‘I still try to be as girly as possible as soon as duty hours are up.’ We heard this student’s struggle to take on the military aspect of her new, professional identity as a military physician, while simultaneously preserving her primary, feminine self.

Her mask visually echoes this division between primary and new identities. It is vertically divided in two, separated by a jagged line. The left side of the mask is painted white with a pink hue of blush on the cheek, a glitter filled eyelid with mascaraed eyelashes, and lips painted bright red. The Given is, here again, the already-known, primary side of femininity. In contrast, the right side of the mask is coloured according the military’s accepted standard of conservative, demure cosmetics use. It is painted in a nude skin tone, with lips coloured naturally, without additional colour. It is decorated only with the Air Force’s emblem branded on the cheek. The New is the yet-to-be-adopted military identity. The split face visually represents identity dissonance, divided between the primary feminine, private identity and the military, professional identity.

At the end of the narrative, we heard this student recognize the dissonance between these two identities. She describes actively deciding to accept both elements (i. e., a feminine woman *and* a military physician) as part of her identity:

Initially I felt that it [femininity] was weakness,but now it’s just a part of me. Ican still be a good officer withouthaving to fundamentally change myself.

The student explains that she ‘wears’ her military bearing in her professional life, but that ‘when my uniform comes off I can still be me.’

In this case, we hear one student’s approach to resolving the irreconcilable aspects of identities that are dissonant: wear one identity at a time.

### Case D

In this case we hear and see a different experience of identity dissonance. While listening for contrapuntal voices, we heard the student distinguish between her pre-military medicine identity (i. e., ‘old me, happy me’) and her identity as a military medical student (i. e., ‘How do you depict constant failure, constant fear, the hollow hopelessness of it all.’) Interestingly, we also heard her describe her current identity as being neither of these, but rather as ‘somewhere between the two: the substrate of who I am.’ In this in-between state, the student describes her identity as a void. She has not grasped the new professional identity, yet she cannot revert to her primary identity. She describes herself as exposed and unmasked; she feels ‘plain, unremarkable, forgettable.’ This student expresses vulnerability in what she writes (e. g., ‘I am falling’) and in the silences of what she is unable to write (i. e., there are no statements of current successes as a military officer, as a medical student, nor in any non – professional identities).

The student describes and paints her mask like armour. Again, the mask is divided vertically with two different paint colours – gold on the left and copper on the right. We considered how these metallic colours of armour could signify a need to guard and protect herself, but could equally signify resiliency. We felt that this resiliency was an important consideration since this mask uses Given/New in a different way than the other masks. Here, the Given is painted with red lines and the downward arrows, which she describes in the narrative as representing her failures in medical school, her fall and the harsh words of the military. The New is painted without angry red lines and is instead decorated with light, soft feathers. In the narrative, she describes this side of the mask as:

Graceful marks for better times. Curves like questions, will I ever get back there? Fly back to the beginning, freedom of flight, soar above my misery.

If the Given space (of what is known and accepted) is where this student visually represents the hard, difficult experience of military medicine training, the New space is where she depicts the unknown future. That future is visually represented with feathers, a powerful contrast to the heavy weight of the Given.

This student’s identity dissonance is represented as unresolved and indeterminate. In analyzing her case, we were unable to hear a strong sense of direction towards professional identity formation.

## Discussion

Each case exhibited strong expressions of the student’s identity dissonance, and provided insights into each trainee’s unique personal experiences of that dissonance at a particular point in their medical school training. Costello defines identity dissonance as an internal conflict between differing aspects of a person’s self – concept [8]. Certainly, we recognized elements of identity dissonance through the cases in this study. This conflict largely presented itself as tension between a primary self that the trainee embodied before coming to USU to train as a military physician, and a new professional self that she needs to integrate. The student creations largely represented negative dissonance – we heard and saw these female students “losing themselves” – rather than positive dissonance – we rarely saw traces of these students ‘finding themselves’ [7, p. 128]. The tensions that were manifested in the cases in this study are consistent with the “disconcert[ion] and unpleasant[ness]” [7, p. 129] that accompanies negative identity dissonance.

According to Vivekananda-Schmidt et al., the development of professional identity is the overlap between the process of learning about themselves and learning about the professional role for which they are training [2]. It requires partially deconstructing the pre-existing identity as part of the process [3]. The pressures associated with this process can lead to feelings of identity dissonance [1]. Fostering self-awareness is imperative in guiding professional identity formation in students [30], and creativity is one avenue students can use to become more familiar with themselves [31].

It is essential to note that professional identity formation is an on-going process. While the masks are fixed objects, identity is not. We chose to conduct our analysis just after students completed an intense period of clerkship activity and a high-stakes examination. The masks in our study, therefore, capture emotions during a point in time when students were transitioning to post-clerkship activities. Recognizing that our students were anecdotally reporting high levels of burnout, we felt that the mask-making activity could serve as an explicit mechanism for students to explore their developing professional identity. The stage of professional identity formation, and the experience of identity dissonance, expressed in these cases were present at a particular moment in time. A longitudinal study of a cohort of students with data collected across their entire educational trajectory could help us understand if identity dissonance is more keenly experienced at some stages of training, or if particular education-related events (e. g., board exams) are related to specific professional identity formation milestones. In such a longitudinal study, having the mask-making students engage in the project as participant researchers would add valuable insights into the intervention’s ability to explore individuals’ professional identity formation. As participant researchers, the students could help the research team explore and understand the professional identity formation issues identified in their masks, and construct support systems that could facilitate the professional identity formation progress and development.

We also chose to focus on representations of identity dissonance. Although not addressed here, we noted other themes through the analysis process that warrant investigation (e. g., role burden). Additionally, we only sampled cases created by women and the analysis team was composed of women. It may be that the participants who made the selected cases were fluent in the creative forms of expression used in this study. We suggest, however, that the success of mask making with wounded warriors shows that a wide range of men and women are skilled in these creative expressions [23]. In accordance with our interpretivist design, our analysis team was actively involved in creating themes and finding insights into professional identity formation and identity dissonance. The goal of this research was not to objectively determine what themes were present in all the masks made by students, but to explore the unique insights we could glean from each case. We aimed to represent the participants’ voice while also acknowledging the active interpretation required to listen.

## Conclusion

Through this study, we have shown that mask making, accompanied by short reflective writing, provided our trainees with an ‘opportunity for projecting, confronting, and exploring aspects of the self’ [32, p. 43]. Through the creative process, the mask-makers externalized feelings of internal conflict by projecting them onto the mask itself [13]. We suggest that mask making and narrative reflection may help to identify students experiencing identity dissonance. Further, we hypothesize that these artistic expressions might be useful for some students as a form of intervention that allows them to become aware of their own identity dissonance. Given the negative ramifications of long-term experiences of identity dissonance, raising student awareness could play an important role in allowing them to recognize the phenomenon in themselves [8]. The insights gained from these masks and narratives may enable educators to tailor additional interventions to each student’s unique needs in terms of developing resilience, wellness and authenticity in the context of individual professional identity.

## Disclaimer

The conclusions made in this study are not the official position of the Uniformed Services University of the Health Sciences or the Department of Defense.
